# A standardized fold change method for microarray differential expression analysis used to reveal genes involved in acute rejection in murine allograft models

**DOI:** 10.1002/2211-5463.12343

**Published:** 2018-01-25

**Authors:** Weichen Zhou, Yi Wang, Masayuki Fujino, Leming Shi, Li Jin, Xiao‐Kang Li, Jiucun Wang

**Affiliations:** ^1^ State Key Laboratory of Genetic Engineering and Ministry of Education Key Laboratory of Contemporary Anthropology Collaborative Innovation Center for Genetics and Development School of Life Sciences and Institutes of Biomedical Sciences Fudan University Shanghai China; ^2^ Department of Computational Medicine & Bioinformatics University of Michigan Ann Arbor MI USA; ^3^ Division of Transplantation Immunology National Research Institute for Child Health and Development Tokyo Japan; ^4^ AIDS Research Center National Institute of Infectious Diseases Tokyo Japan

**Keywords:** gene expression, microarray analysis, murine transplantation model, standardized fold change

## Abstract

Murine transplantation models are used extensively to research immunological rejection and tolerance. Here we studied both murine heart and liver allograft models using microarray technology. We had difficulty in identifying genes related to acute rejections expressed in both heart and liver transplantation models using two standard methodologies: Student's *t* test and linear models for microarray data (Limma). Here we describe a new method, standardized fold change (SFC), for differential analysis of microarray data. We estimated the performance of SFC, the *t* test and Limma by generating simulated microarray data 100 times. SFC performed better than the *t* test and showed a higher sensitivity than Limma where there is a larger value for fold change of expression. SFC gave better reproducibility than Limma and the *t* test with real experimental data from the MicroArray Quality Control platform and expression data from a mouse cardiac allograft. Eventually, a group of significant overlapping genes was detected by SFC in the expression data of mouse cardiac and hepatic allografts and further validated with the quantitative RT‐PCR assay. The group included genes for important reactions of transplantation rejection and revealed functional changes of the immune system in both heart and liver of the mouse model. We suggest that SFC can be utilized to stably and effectively detect differential gene expression and to explore microarray data in further studies.

**Abbreviations**
FNR: false negative rate
FPR: false positive rate
Limma: linear models for microarray data
MAQC: MicroArray Quality Control
POD: post‐operative day
qRT‐PCR: quantitative RT‐PCR
SFC: standardized fold change

At the stage of organ failure, organ transplantation is a life‐saving medical procedure, though it still has some problems, e.g. transplant rejection and the requirement for life‐long immunosuppressive drugs. Transplantation models without immunosuppression are important and the mechanisms of rejection and tolerance in these models need to be revealed.

Microarray technology is well established and widely used, providing a picture of gene expression or RNA profiling in different tissues 1. To identify differential expression, Student's *t* test and linear models for microarray data (Limma) are two popular choices 2, 3, 4. The *t* test utilizes information for all the samples (or standard deviations) in one microarray probe and is conducted independently among different probes 4, while Limma uses the empirical Bayesian approach of shrinkage of the estimated sample variances towards a pooled estimate. The information (means and standard deviations) from all the probes in a replicate set of experiments is combined and used at the level of one probe to detect differential expression in Limma 2.

In the present study, we established murine heart and liver allograft models and used microarray technology to reveal the significant genes that related to transplant rejection. By using the *t* test and Limma, no significant intersecting genes were obtained in these models. Therefore, we developed a new method, named standardized fold change (SFC), to detect differential expression by taking information from the neighbors of one probe with an adjustable bin size. To compare SFC with the *t* test and Limma, we generated a simulated data set to estimate the performance and used the real experimental datasets from the MicroArray Quality Control (MAQC) platform and the transplantation model to estimate the reproducibility. We concluded that SFC can be applied as a new and effective approach to detect differential expression and contribute more reliable results in microarray studies. Then, SFC reported a set of significant genes from expression data from the murine heart and liver allograft, and we further validated them by qRT‐PCR. Gene expression changes revealed functional reactions and pathway activities in the early stage of allograft in both heart and liver.

## Materials and methods

### Animals

Male B10.BR (BR, H‐2k), B10.D2 (D2, H‐2d), C57BL/10 (B10, H‐2b) and CBA (H‐2k) mice (weighing 25–30 g) were purchased from the Shizuoka Laboratory Animal Center (Shizuoka, Japan) and housed and cared for in agreement with the guidelines of the Institutional Animal Care and Use Committee and the National Research Institute (Japan) for Child Health and Development guidelines on laboratory animal welfare. The Committee on the Care and Use of Laboratory Animals at the National Research Institute accepted the experimental protocol for Child Health and Development (Permission no.: 2002‐003). All surgical procedures were conducted under anesthesia with isoflurane/oxygen, and all attempts were made to minimize suffering.

### Transplantation and RNA extraction

Cardiac transplantation was performed from a sex‐matched B10 donor to a CBA recipient by microsurgical techniques. Intra‐abdominal vascularized heterotopic mouse cardiac transplantation was performed 5. The cardiac graft survival was determined using daily palpation of the recipient's abdomen. Three case samples on the fifth day were obtained. BR mice were used as donors and D2 mice were used as recipients in the orthotopic hepatic transplantation. We performed transplantation surgery on the mice 6 in which for orthotopic liver transplantation, BR mice were used as donors and D2 mice were used as recipients. We subsequently transplanted the liver into the recipient mice using the cuff technique 6. Grafts were harvested at post‐operative day 5 (POD5) or at POD8 after transplantation and were submerged in RNAlater® stabilization solution (Life Technologies, Carlsbad, CA, USA) for freezing. Total RNA was extracted from frozen tissue samples using ISOGEN (NipponGene, Tokyo, Japan). We also designed control groups of three normal cardiac tissues and three hepatic tissues.

### Standardized fold change method

The probe signals from microarray data were firstly natural log‐transformed and then manipulated with quantitative normalization. To assess the differential expressions among cases and controls, the statistic SFC is defined as:

(1) $${\text{SFC}}_{i}=\frac{T-C}{\text{STDEV}(T-C)}=\frac{T_{i}-C_{i}}{\sqrt{\text{Var}\left(T-C\right)}}=\left(\frac{\text{Median}\left(t_{1},t_{2}\ldots t_{i}\right)-\text{Median}\left(c_{1},c_{2},\ldots c_{i}\right)}{\sqrt{\text{Median}\left({\left(T_{i-b/2}-C_{i-b/2}\right)}^{2},\ldots {\left(T_{i}-C_{i}\right)}^{2},\ldots {\left(T_{i+b/2}-C_{i+b/2}\right)}^{2}\right)/0.455}}\right).$$

For the variance of each probe, we ranked all probes by the mean values of signals from all samples and then took the median value of its *b* nearest neighbors as the variance, where the default bin size of *b* here is 1000. The SFC software now implements this algorithm in the Linux system and can be found at https://github.com/WeichenZhou/SFC.

### Simulation data study

We generated the simulated data from simple formulas with the Gaussian noise (mean = 0, variance = 1) as a default distribution for gene expression data 7. The control and case samples in the null hypothesis are shown as follows:

$$\text{H0 control}:y_{0}=x_{0}+N\left(0,1\right)\left[\sqrt{(kx_{0})+1}\right]$$

(2) $$\text{H0 case}:y_{0}^{\prime }=\left(1+\theta_{0}\right)x_{0}^{\prime }+N\left(0,1\right)\left[\sqrt{(kx_{0}^{\prime })+1}\right]$$

where θ represented the differential expression underlying cases versus controls and we defined θ_0_ as 0% and *k* is 1. The control and case samples in the alternative hypothesis are shown as follows:

$$\text{H1 control}:y_{1}=x_{1}+N\left(0,1\right)\left[\sqrt{(kx_{1})+1}\right]$$

(3) $$\text{H1 case}:y_{1}^{\prime }=\left(1+\theta_{1}\right)x_{1}^{\prime }+N\left(0,1\right)\left[\sqrt{(kx_{1}^{\prime })+1}\right].$$

We defined θ_1_ as 10%, 25% and 50%, respectively. The size of real positive calls consists of 1%, 5% and 10% of the whole simulated data, respectively. Following these, a 100‐time simulation was conducted to assess the false positive rate (FPR) and the false negative rate (FNR).

### MAQC data and the reproducibility analysis

The MAQC project was developed by the US Food and Drug Administration (FDA) to provide standards and quality control metrics and involved six centers [Applied Biosystems (Thermo Fisher Scientific, Waltham, MA, USA), Affymetrix (Santa Clara, CA, USA), Agilent Technologies (Santa Clara, CA, USA), GE Healthcare (Chicago, IL, USA), Illumina (San Diego, CA, USA) and Eppendorf (Hamburg, Germany)] that are major providers of microarray platforms and RNA samples 1, 8. The reproducibility of the top 100 and 1000 significant genes was estimated inter‐ and intra‐platform by the three statistical methods, and heatmaps were drawn with the matrix of each batch. For the expression data from the mouse transplant model, we picked up two out of three cases and controls to build one batch and made a 9 × 9 matrix heatmap to estimate the reproducibility. The significance level of mouse microarray data was 0.05.

### Application on mouse transplantation data

We detected differential expression of genes between cases and controls in three phases: POD5 of cardiac transplantation, POD5 of hepatic transplantation and POD8 of hepatic transplantation. All *P*‐values from expression data were adjusted by the Bonferroni correction. After getting all significant probes from SFC, we converted the probe level significance to gene level using an annotation file. Venn diagrams showed the significant genes with differential expression. Pathway and gene ontology (GO) enrichment analyses were performed by using the Database for Annotation, Visualization and Integrated Discovery (DAVID; http://david.abcc.ncifcrf.gov/) with the Bonferroni correction‐adjusted *P*‐values < 0.05 9. Mouse transplantation data have been deposited in NCBI's Gene Expression Omnibus 10 and are accessible through GEO Series accession no. GSE89340. All data were conducted by quantile normalization before processing by different methods. Limma can be found as the R package limma 2, 3 and the heatmaps were created by gplots. All R packages can be downloaded from Bioconductor (www.bioconductor.org).

### Quantitative RT‐PCR (qRT‐PCR)

The RNA was reverse‐transcribed to cDNA using a PrimeScript® RT Reagent Kit (Takara Bio, Shiga, Japan) as described previously 11. The sequences used in our study are shown in Table S3. Quantitative RT‐PCR (qRT‐PCR) was performed using a SYBR Green system on the Applied Biosystems PRISM7700 instrument (Thermo Fisher Scientific), and experiments were conducted using 0.4 μm of each primer in a final reaction volume of 20 μL of KAPA SYBR® FAST qPCR kit (Kapa Biosystems, Cape Town, South Africa). The PCR cycling conditions were as follows: 95 °C for 30 s, and 50 cycles of 95 °C for 5 s, 60 °C for 1 min. The normalized threshold cycle (*C*_t_) value of each gene was obtained by subtracting the *C*_t_ value obtained for 18S rRNA. The cardiac mRNA levels were analyzed on POD5. Figure 4 indicates the number of copies of each of the three representative mRNAs measured in the syngeneic grafts or allografts obtained from three individuals. The relative amount of each mRNA was normalized to that of 18S rRNA. All experiments were analyzed in three mice per time point and expressed as the mean ± SEM. The significance level was set as *P *<* *0.05 compared with syngeneic grafts on day 5.

## Results

### The SFC method

We observed that the distribution of the mean value and variance of one probe signal is non‐linear (Fig. S1). The information from neighboring probes can usually be borrowed to improve the statistical power 2. SFC was introduced to estimate variance for each probe, rather than obtaining this from all samples; it takes information from the neighbors of that probe with an adjustable bin size *b*. As we set up the default value of *b* as 1000, the variance of cases and controls in one probe can be obtained by calculating the median for those probes separately. Eventually, by following Eqn (1), we can obtain the statistic SFC for every probe, and the *P*‐value can be further estimated based from these.

### SFC had a better sensitivity and specificity based on simulation data

We investigated the FPR and the FNR of the three methods under the null hypothesis and alternative hypothesis. As indicated in Eqn (2), signals of the null hypothesis were generated by a simple formula, *y *=* x*, with the Gaussian noise added. The basic formulas are adjustable with the parameters *k*. The signals of the alternative hypothesis were described by Eqn (3), with variable values of θ and the portion of real positive calls. We calculated the FPR and FNR for every different θ and portion of real positive calls with a 0.05 significance threshold and 100‐times simulation (Table 1).

**Table 1:** Evaluation of the three methods with *P *<* *0.05

	*t* test	Limma	SFC	θ
H0				
FPR (%)	5.043	5.222	5.694	
FNR (%)	0.000	0.000	0.000	
Calls in total (%)	5.043	5.222	5.694	
H1: simulated real positive calls = 1%				
FPR (%)	6.043	5.455	5.350	10%
	8.763	6.306	5.038	25%
	14.255	8.600	3.990	50%
FNR (%)	6.825	15.367	6.958	10%
	0.783	1.933	0.058	25%
	0.808	0.025	0.000	50%
Calls in total (%)	6.908	6.240	6.220	10%
	9.661	7.217	5.980	25%
	15.098	9.507	4.943	50%
H1: simulated real positive calls = 5%				
FPR (%)	13.306	7.987	3.616	10%
	32.978	17.856	1.818	25%
	52.026	34.301	1.057	50%
FNR (%)	6.942	15.283	8.224	10%
	0.492	2.108	0.075	25%
	0.699	0.020	0.000	50%
Calls in total (%)	17.290	11.820	8.020	10%
	36.301	21.854	6.714	25%
	54.388	37.5817	5.999	50%
H1: simulated real positive calls = 10%				
FPR (%)	27.850	13.782	1.615	10%
	56.305	35.345	0.626	25%
	73.170	57.081	0.266	50%
FNR (%)	7.282	15.334	9.830	10%
	0.551	2.042	0.277	25%
	0.652	0.019	0.000	50%
Calls in total (%)	34.336	20.870	10.470	10%
	60.619	41.606	10.535	25%
	75.787	61.371	10.238	50%

**Figure 1:**
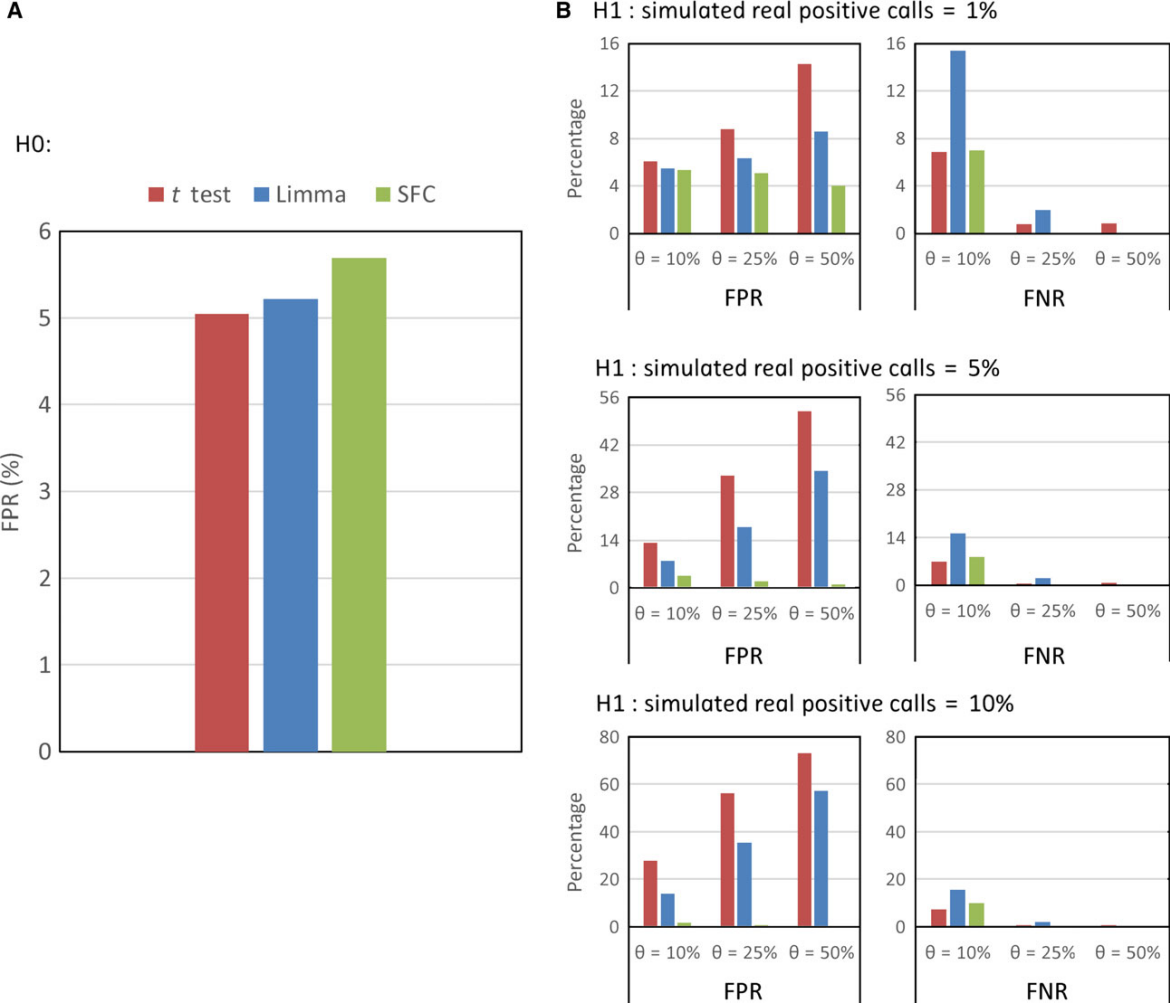
Bar graphs of FPR and FNR from the three methods under the null hypothesis (H0) and the alternative hypothesis (H1). (A) FPR under the null hypothesis (FN = 0). (B) FPR and FNR under different alternative hypotheses, in which θ is equal to 10%, 25% and 50% and the simulated real positive calls are 1%, 5% and 10% of the whole simulated data, respectively. The significance threshold is 0.05.

Under the null hypothesis, the rates of the three methods are all near the significance threshold between 5% and 6% (Fig. 1A). Under the alternative hypothesis, SFC had a better performance for FPR than the other two methods generally (Fig. 1B). With an increasing θ and portion of real positive calls, the FPR of SFC showed a decreasing bias, whereas Limma and the *t* test showed a positive bias with these parameters (Table 1). For the FNR, as the θ and portion of real positive calls increased, Limma showed a faster decline than the *t* test, while SFC had a lower FNR than Limma and performed better with larger θ and portion of real positive calls. Interestingly, SFC shows a relatively small number of calls (from 4.9% to 10.5%, Table 1), while Limma and the t test calls a larger set in this situation. In sum, comparing with Limma and the *t* test at the significance threshold of 0.05, SFC had a better sensitivity and specificity, especially with a larger value of differential expression fold change (θ = 50%).

### Reproducibility of SFC is better than Limma and the t test based on MAQC and mouse transplantation data

Reproducibility is an indispensable estimator for the experiments and algorithms 12, 13. We chose both the MAQC dataset and the mouse cardiac transplantation data to assess the reproducibility of SFC, Limma and the *t* test.

**Figure 2:**
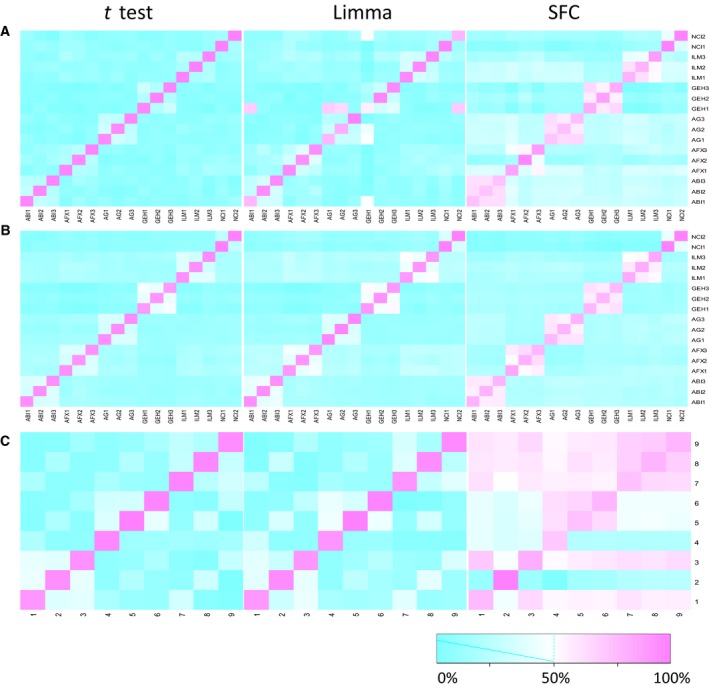
Heatmaps of reproducibility analysis. (A) Reproducibility of top 100 significant genes by *t* test, Limma and SFC based on MAQC data. (B) Reproducibility of top 1000 significant genes by the three methods based on MAQC data. (C) Reproducibility of significant genes by the three methods based on pairwise analysis of data from the mouse cardiac graft model.

We calculated the reproducibility of the top 100 and top 1000 genes for MAQC by using the three methods. For the interplatform comparison, the heatmap shows that SFC performed a better reproducibility than Limma and the *t* test among six platforms when detecting both the top 100 and the top 1000, while for intra‐platform reproducibility, all three methods did not perform well in detecting either the top 100 or the top 1000 significant genes (Fig. 2A,B). The same operations were conducted in the mouse cardiac transplantation data, where SFC also showed a better performance than the others (Fig. 2C). Therefore, according to better performances of reproducibility for both the MAQC data and the mouse transplantation data, SFC is more stable than Limma or the *t* test.

### Intersected significances from mouse transplantation data were found by SFC and validated by qRT‐PCR

We further utilized the three methods to analyze the mouse organ transplantation data and validated the results. After the experimental process generating CEL files from mouse tissues, we conducted these methods on the expression data of POD5 of cardiac transplantation and POD5 and POD8 of hepatic transplantation.

**Figure 3:**
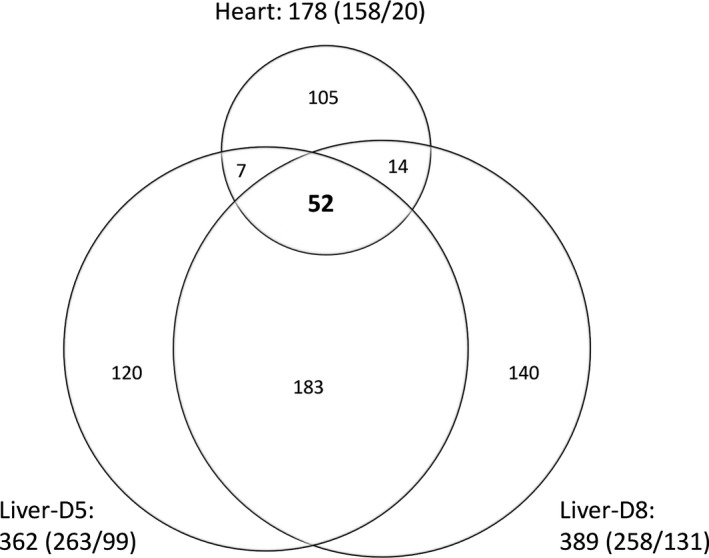
Venn diagram of significant genes analyzed by SFC with the level of significance set at *P < *0.05 after the Bonferroni correction. The overall numbers of significant genes in three phases are shown outside, which are followed by numbers in parentheses showing the counts of overexpressed genes versus underexpressed ones. The circle at the top represents POD5 for heart; the circle at the bottom left represents POD5 for liver and the circle at the bottom right represents POD8 for liver.

According to SFC, 178 significant genes were differentially expressed in the cardiac allografts compared with isografts, including 158 overexpressed genes and 20 underexpressed genes (Fig. 3). There were also 362 genes (263 overexpression and 99 underexpression) having significantly different expression in the hepatic POD5 allografts compared with isografts, and 389 genes (258 overexpression and 131 underexpression) having significantly different expression in the hepatic POD8 allografts compared with isografts. Based on these, an intersection of these three groups was obtained that included 52 important genes, in which they are all overexpressed for cardiac transplantation and 51 overexpressed and one underexpressed for hepatic transplantation (Fig. 3). At the same time, the calling sets of significant genes underlying Limma and the *t* test (Fig. S4A,B) showed no intersecting ones.

**Figure 4:**
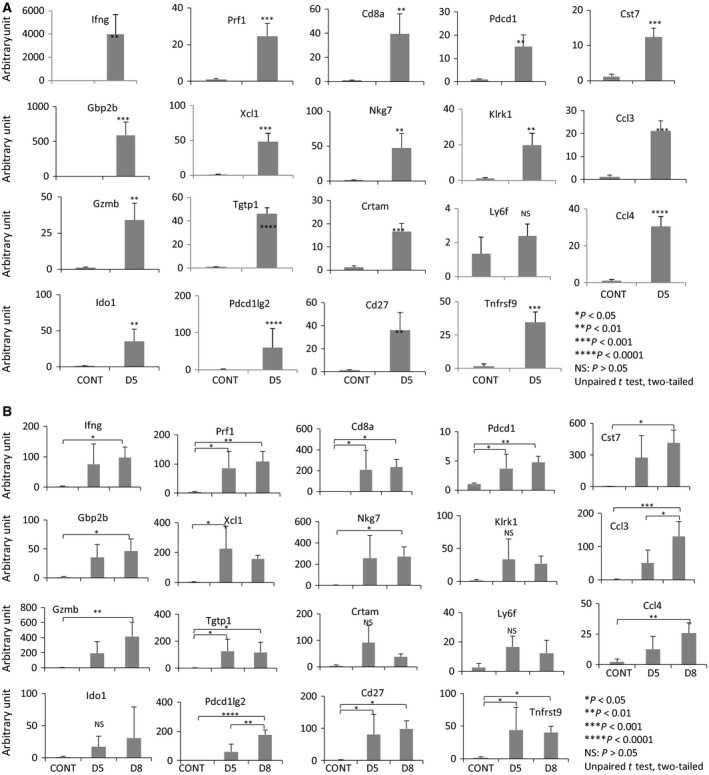
Validation of the microarray data using a qRT‐PCR assay in the mouse cardiac graft model and hepatic graft model. (A) Cardiac mRNA levels analyzed on POD5, indicating the values of mRNAs measured in the syngeneic grafts (CONT) or allografts (D5) obtained from three individuals. (B) Hepatic mRNA levels analyzed on POD5 and POD8, indicating the value of mRNAs measured in the syngeneic grafts (CONT) or allografts (D5 or D8) obtained from three individuals. A two‐tailed unpaired *t* test was used to calculate *P*‐values comparing syngeneic grafts with allografts.

We further performed qRT‐PCR for the calls derived from SFC to validate the fold change of the mRNA expression. Nineteen mRNAs, which were upregulated in both the cardiac and the hepatic allografts compared with isografts, were randomly selected (Tables 2 and S3). Being consistent with the results of microarray, a significantly higher amount of mRNA expression was detected in allografts versus isografts in cardiac (Fig. 4A) and hepatic (Fig. 4B) allografts.

**Table 2:** List of validated genes

Accession no.	Gene	Gene name	Fold‐heart	Fold‐liver‐D5	Fold‐liver‐D8
NM_008337	*Ifng*	Interferon gamma	1593.863	54.675	72.591
NM_010259	*Gbp2b*	Guanylate binding protein 2b	1263.049	12.951	18.460
NM_013542	*Gzmb*	Granzyme B	185.351	147.035	114.736
NM_008324	*Ido1*	Indoleamine‐2,3‐dioxygenase 1	103.729	38.474	47.050
NM_011073	*Prf1*	Perforin 1 (pore forming protein)	99.539	38.016	37.767
NM_008510	*Xcl1*	Chemokine (C motif) ligand 1	82.096	27.777	26.918
NM_011579	*Tgtp1*	T cell specific GTPase 1	76.367	33.074	59.197
NM_021396	*Pdcd1lg2*	Programmed cell death 1 ligand 2	74.231	14.479	41.463
NM_001081110	*Cd8a*	CD8 antigen, alpha chain	60.400	33.458	32.012
NM_024253	*Nkg7*	Natural killer cell group 7 sequence	47.828	38.247	30.322
NM_019465	*Crtam*	Cytotoxic and regulatory T cell molecule	46.089	26.296	15.863
NM_001033126	*Cd27*	CD27 antigen	33.240	39.830	41.565
NM_008798	*Pdcd1*	Programmed cell death 1	29.391	74.356	69.542
NM_033078	*Klrk1*	Killer cell lectin‐like receptor subfamily K, member 1	28.611	18.487	16.631
NM_008530	*Ly6f*	Lymphocyte antigen 6 complex, locus F	27.006	56.930	29.637
NM_011612	*Tnfrsf9*	Tumor necrosis factor receptor superfamily, member 9	26.947	30.625	29.872
NM_009977	*Cst7*	Cystatin F (leukocystatin)	25.625	26.383	30.931
NM_011337	*Ccl3*	Chemokine (C‐C motif) ligand 3	21.102	47.883	82.279
NM_013652	*Ccl4*	Chemokine (C‐C motif) ligand 4	19.907	35.686	56.794

## Discussion

Microarray is widely used and accepted as a stable, well established and less costly technology to investigate gene expression data 1, 8, 14, 15. In this study based on microarray data, we established a novel method, SFC, to detect differential expression and compared it with the *t* test and Limma. According to Eqn (1), the parameter *b* can be adjusted to control the nearby number of probes, which contribute the variance of the central probe. We set 1000 as default, and users are able to customize this value based on a different number size of microarray probe. For the simulation data, the parameter configurations (θ and *k*) of the null hypothesis and alternative hypothesis also can be adjusted (Eqns (2) and (3)) 7. Moreover, we calculated the FPR and FNR based on different significance levels (*P *=* *0.01 and 0.001) for different values of θ and *k*. With a more stringent significance level (0.05, 0.001), the FPRs were decreased while the FNRs were increased, which was observed by all three methods (Figs 1, S2 and S3, Tables 1, S1 and S2). Notably, when *P *=* *0.001, the *t* test gave a high FPR (48%, θ = 50%, and the true positive gene percentage was 10%) and Limma performed with a high FNR (sometimes more than 90%). This suggests the *t* test will give more positive hits with a high FPR, while Limma will report fewer hits to reduce the FPR but miss some true positive ones. Importantly, SFC can give a good balance of FPR and FNR, and perform well for both FPR and FNR with a stringent significance level.

Statistical correction (e.g. the Bonferroni correction) is often introduced for multiple comparisons to adjust the *P*‐value and control the false discovery rate 16. We also analyzed the mouse transplantation data by the other two methods (Limma and the *t* test) with different significance levels (*P *=* *0.05, 0.001 and 0.05 with the Bonferroni correction). Limma and the *t* test had a large number of positive hits when the *P*‐value was 0.05 in three phases (Figs S5 and S6). When the level of significance was *P *<* *0.001, the positive hits by Limma and the *t* test decreased a lot while by SFC the number stayed relatively stable. When the *P*‐value was stringent at 0.05 with the Bonferroni correction (Figs 3 and S4), SFC still reported 52 significances overlapping with three phases, but Limma and the *t* test showed no overlapping significance. The results of the *t* test showed no shared significance with SFC. Intriguingly, in 67 significances for cardiac POD5 reported by Limma (Fig. S4), 30 genes showed in the cardiac POD5 result for SFC, and 16 showed in the 52 significances. Besides, for hepatic POD5 and POD8 by Limma, 4 out of 7 (POD5) and 19 out of 36 (POD8) significant genes were observed in the corresponding results of SFC, and 2 out of 5 (overlapping in POD5 and POD8) significant genes appear in the 52 genes from SFC. As 19 of 52 genes from SFC were randomly selected and all passed the validation of qRT‐PCR, these results indicated that SFC gave a more stable result than the *t* test and Limma.

We therefore investigated the functions of these 52 genes (Table S4), revealing the most significant pathways were graft‐versus‐host disease (mmu05332) and allograft rejection (mmu05330). Moreover, immune system response (e.g. mmu04612, mmu04660, GO: 0006955) and positive regulation (e.g. GO: 0050863, GO: 0051249, GO: 0050870) were also activated. All these enrichment analyses indicated a reaction of transplantation rejection *in vivo* and functional changes of the immune system both at the cardiac and at the hepatic level after 5 days of allografts 6, 17, 18.

In conclusion, based on the quality control experimental data and simulated data, SFC performed better than Limma and much better than the *t* test by using the nearby information of one probe in pooled probes. We utilized SFC for the real data of mouse transplantation models, and it reported a more stable and convincing set with 52 significant genes, revealing insights into pathway and gene expression changes after both cardiac and hepatic allografts. Nineteen genes were further randomly picked up and validated by qRT‐PCR. We suggest SFC is a new and effective approach that can detect differential expression and help to obtain more reliable information in microarray studies.

## Author contributions

WZ, YW, XL and JW designed the project. LS supported the MAQC data, and MF and XL supported the mouse model and validations. WZ carried out the analysis and simulations. WZ, XL and JW wrote the manuscript. LJ, XL and JW contributed to the final revision the paper. All authors read and approved the final manuscript.

## Supporting information


**Fig. S1.** Distribution of mean and variance of sample microarray signals in each probe derived from the MAQC data.

**Fig. S2.** Bar graphs of FPR and FNR from the three methods under the null hypothesis (H0) and the alternative hypothesis (H1) with the level of significance set at *P *<* *0.01.

**Fig. S3.** Bar graphs of FPR and FNR from the three methods under the null hypothesis (H0) and the alternative hypothesis (H1) with the level of significance set at *P *<* *0.001.

**Fig. S4.** Venn diagrams of significant gene numbers analyzed by the *t* test and Limma with the level of significance set at *P *<* *0.05 after the Bonferroni correction.

**Fig. S5.** Venn diagrams of significant gene numbers analyzed by the *t* test, Limma and SFC with the level of significance set at *P *<* *0.05.

**Fig. S6.** Venn diagrams of significant gene numbers analyzed by the *t* test, Limma and SFC with the level of significance set at *P *<* *0.001.

**Table S1.** Evaluation of three methods with the level of significance set at *P *<* *0.01.
**Table S2.** Evaluation of three methods with the level of significance set at *P *<* *0.001.
**Table S3.** Primer sequences for qRT‐PCR.

**Table S4.** GO term and pathway enrichment analysis based on the 52 significant genes.

## References

[1] MAQC Consortium , Shi L , Reid LH , Jones WD , Shippy R , Warrington JA , Baker SC , Collins PJ , de Longueville F , Kawasaki ES , *et al* (2006) The MicroArray Quality Control (MAQC) project shows inter‐ and intraplatform reproducibility of gene expression measurements. Nat Biotechnol 24, 1151–1161. 16964229 10.1038/nbt1239 PMC3272078

[2] Smyth GK (2004) Linear models and empirical bayes methods for assessing differential expression in microarray experiments. Stat Appl Genet Mol Biol 3, Article 3. 10.2202/1544-6115.1027 16646809

[3] Ritchie ME , Phipson B , Wu D , Hu Y , Law CW , Shi W and Smyth GK (2015) limma powers differential expression analyses for RNA‐sequencing and microarray studies. Nucleic Acids Res 43, e47. 25605792 10.1093/nar/gkv007 PMC4402510

[4] Rice JA (2007) Mathematical Statistics and Data Analysis, 3rd edn Thomson/Brooks/Cole, Belmont, CA.

[5] Hou J , Zhang Q , Fujino M , Cai S , Ito H , Takahashi K , Abe F , Nakajima M , Tanaka T , Xu J *et al* (2015) 5‐Aminolevulinic acid with ferrous iron induces permanent cardiac allograft acceptance in mice via induction of regulatory cells. J Heart Lung Transplant 34, 254–263. 25455753 10.1016/j.healun.2014.09.037

[6] Morita M , Chen J , Fujino M , Kitazawa Y , Sugioka A , Zhong L and Li XK (2014) Identification of microRNAs involved in acute rejection and spontaneous tolerance in murine hepatic allografts. Sci Rep 4, 6649. 25323448 10.1038/srep06649 PMC5377586

[7] Wang Y , Li Y , Cao H , Xiong M , Shugart YY and Jin L (2015) Efficient test for nonlinear dependence of two continuous variables. BMC Bioinformatics 16, 260. 26283601 10.1186/s12859-015-0697-7 PMC4539721

[8] Shi L , Campbell G , Jones WD , Campagne F , Wen Z , Walker SJ , Su Z , Chu TM , Goodsaid FM , Pusztai L , *et al* (2010) The MicroArray Quality Control (MAQC)‐II study of common practices for the development and validation of microarray‐based predictive models. Nat Biotechnol 28, 827–838. 20676074 10.1038/nbt.1665 PMC3315840

[9] Dennis G Jr , Sherman BT , Hosack DA , Yang J , Gao W , Lane HC and Lempicki RA (2003) DAVID: database for annotation, visualization, and integrated discovery. Genome Biol 4, P3. 12734009 PMC193660

[10] Edgar R , Domrachev M and Lash AE (2002) Gene expression omnibus: NCBI gene expression and hybridization array data repository. Nucleic Acids Res 30, 207–210. 11752295 10.1093/nar/30.1.207 PMC99122

[11] Nishio Y , Fujino M , Cai S , Kitajima Y , Saito T , Tsumura H , Ito M , Ito Y , Nagahara Y and Li XK (2016) Impaired CD98 signaling protects against graft‐versus‐host disease by increasing regulatory T cells. Transpl Immunol 35, 34–39. 26836475 10.1016/j.trim.2016.01.005

[12] Vaux DL , Fidler F and Cumming G (2012) Replicates and repeats–what is the difference and is it significant? A brief discussion of statistics and experimental design EMBO Rep 13, 291–296. 22421999 10.1038/embor.2012.36 PMC3321166

[13] Fomel S and Claerbout JF (2009) Guest editors’ introduction: reproducible research. Comput Sci Eng 11, 5–7.

[14] Barrett T and Edgar R (2006) Mining microarray data at NCBI's Gene Expression Omnibus (GEO)*. Methods Mol Biol 338, 175–190. 16888359 10.1385/1-59745-097-9:175 PMC1619899

[15] Roy NC , Altermann E , Park ZA and McNabb WC (2011) A comparison of analog and Next‐Generation transcriptomic tools for mammalian studies. Brief Funct Genomics 10, 135–150. 21389008 10.1093/bfgp/elr005

[16] Cui X and Churchill GA (2003) Statistical tests for differential expression in cDNA microarray experiments. Genome Biol 4, 210. 12702200 10.1186/gb-2003-4-4-210 PMC154570

[17] Chen X , Chang CH , Stein R , Cardillo TM , Gold DV and Goldenberg DM (2013) Prevention of acute graft‐versus‐host disease in a xenogeneic SCID mouse model by the humanized anti‐CD74 antagonistic antibody milatuzumab. Biol Blood Marrow Transplant 19, 28–39. 23025988 10.1016/j.bbmt.2012.09.015

[18] Fujiwara H , Maeda Y , Kobayashi K , Nishimori H , Matsuoka K , Fujii N , Kondo E , Tanaka T , Chen L , Azuma M *et al* (2014) Programmed death‐1 pathway in host tissues ameliorates Th17/Th1‐mediated experimental chronic graft‐versus‐host disease. J Immunol 193, 2565–2573. 25080485 10.4049/jimmunol.1400954

