# Supplementary figures and images for: A standardized fold change method for microarray differential expression analysis used to reveal genes involved in acute rejection in murine allograft models
Source: FEBS Open Bio. 2018 Jan 25;8(3):481–90. doi: 10.1002/2211-5463.12343 (PMC5832988; doi:10.1002/2211-5463.12343)

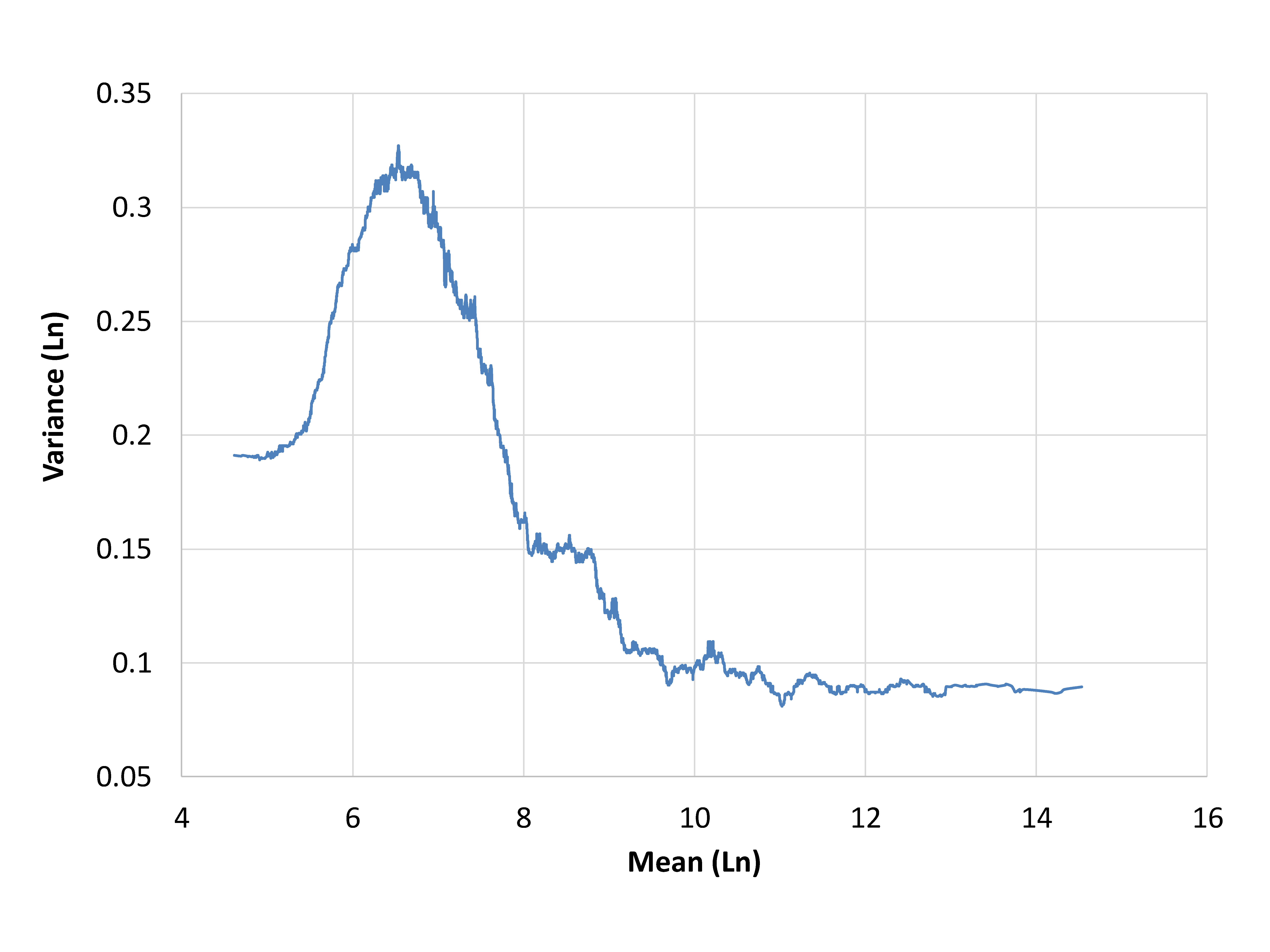

Supplement: Supplementary file 1 — Fig. S1. Distribution of mean and variance of sample microarray signals in each probe derived from the MAQC data. [file FEB4-8-481-s001.tiff]

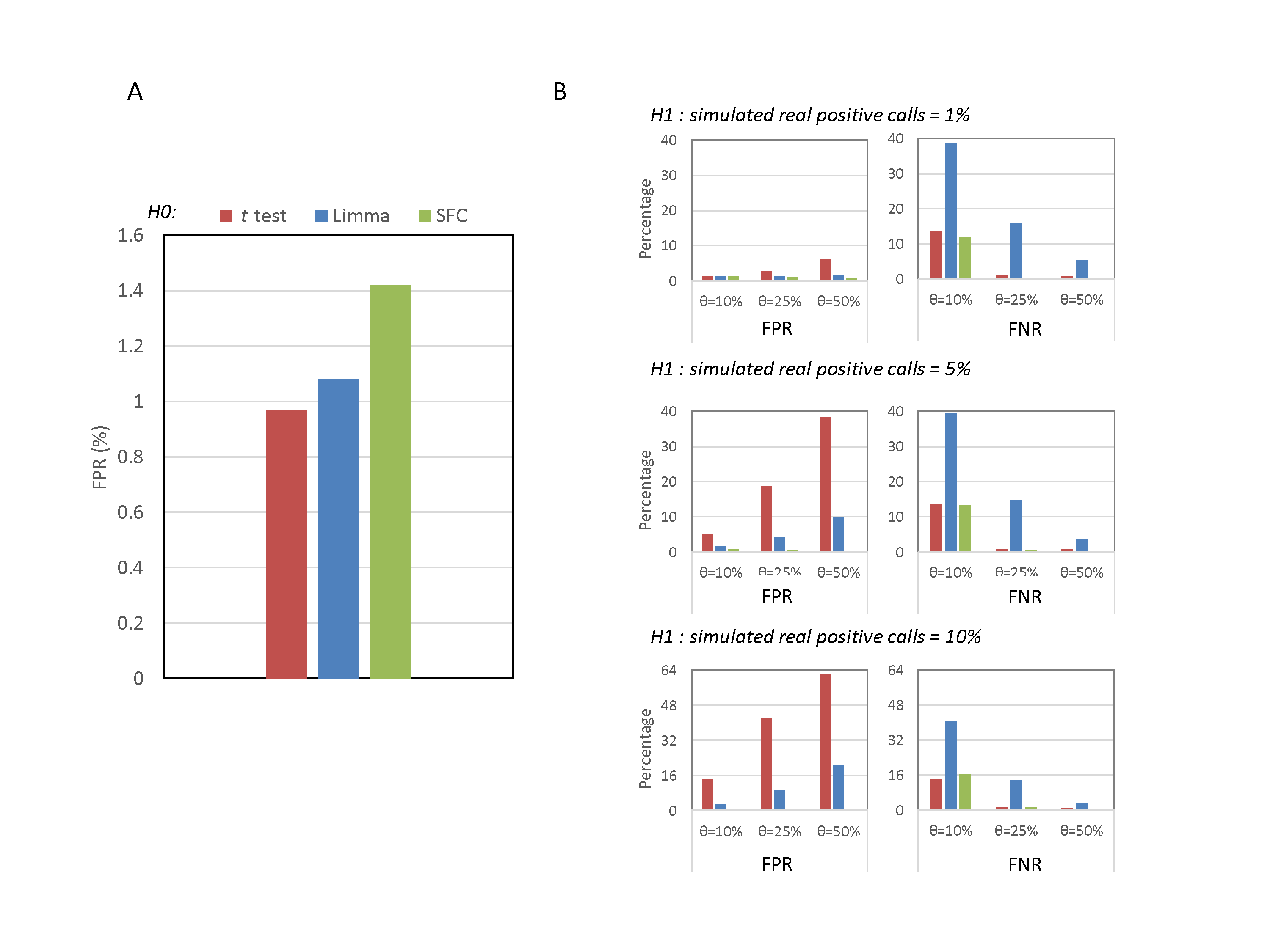

Supplement: Supplementary file 2 — Fig. S2. Bar graphs of FPR and FNR from the three methods under the null hypothesis (H0) and the alternative hypothesis (H1) with the level of significance set at P < 0.01. [file FEB4-8-481-s002.tiff]

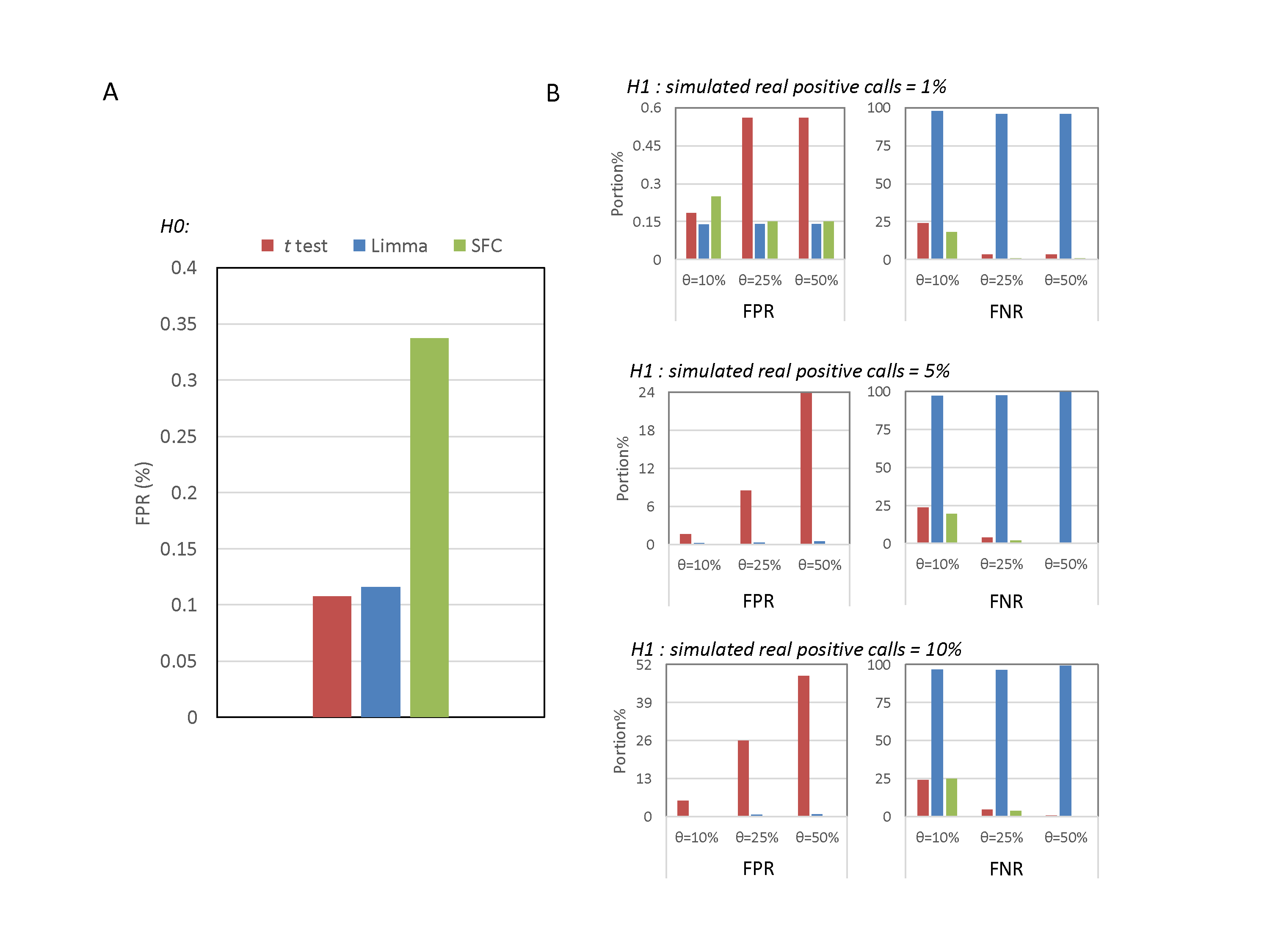

Supplement: Supplementary file 3 — Fig. S3. Bar graphs of FPR and FNR from the three methods under the null hypothesis (H0) and the alternative hypothesis (H1) with the level of significance set at P < 0.001. [file FEB4-8-481-s003.tiff]

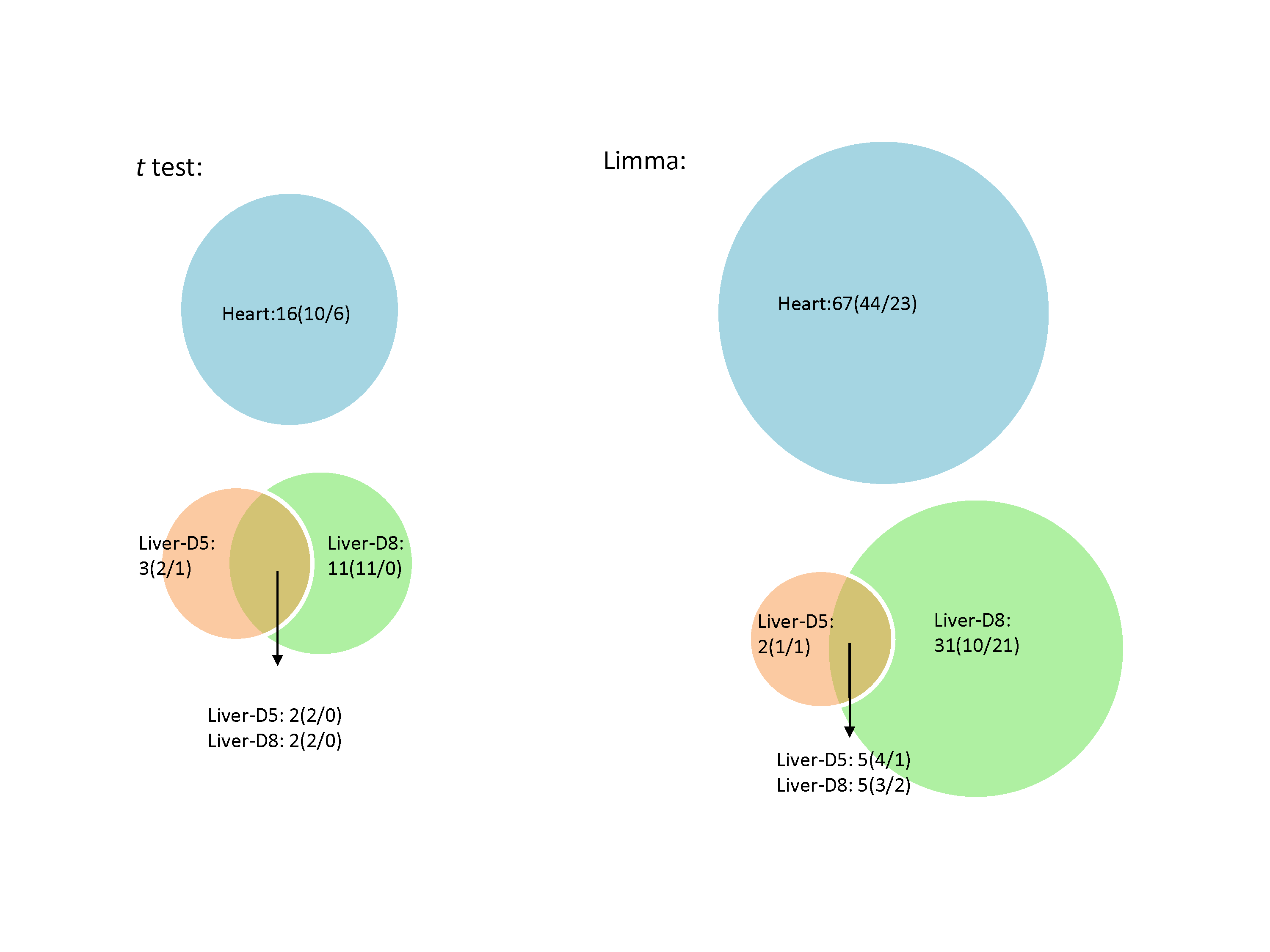

Supplement: Supplementary file 4 — Fig. S4. Venn diagrams of significant gene numbers analyzed by the t test and Limma with the level of significance set at P < 0.05 after the Bonferroni correction. [file FEB4-8-481-s004.tiff]

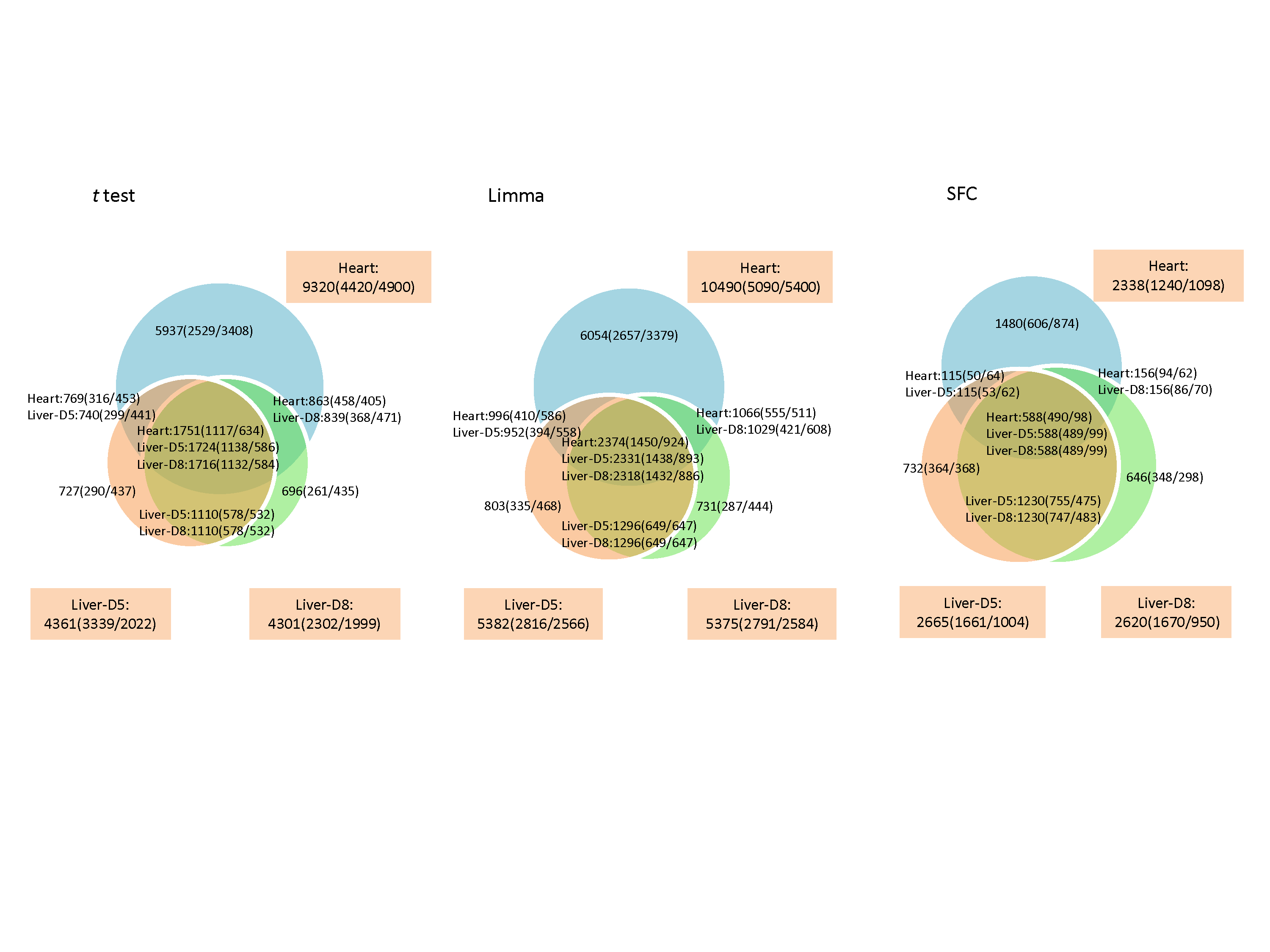

Supplement: Supplementary file 5 — Fig. S5. Venn diagrams of significant gene numbers analyzed by the t test, Limma and SFC with the level of significance set at P < 0.05. [file FEB4-8-481-s005.tiff]

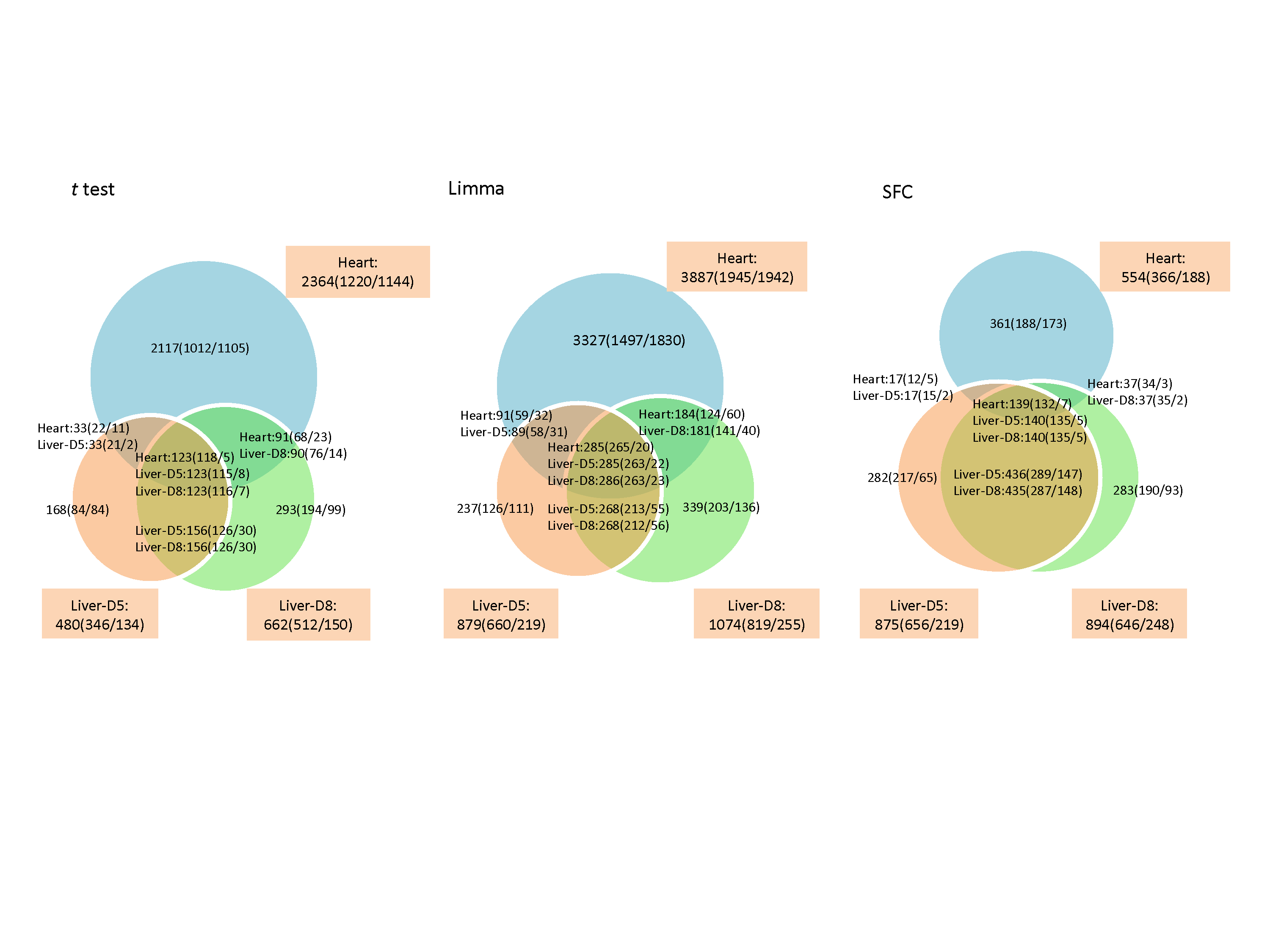

Supplement: Supplementary file 6 — Fig. S6. Venn diagrams of significant gene numbers analyzed by the t test, Limma and SFC with the level of significance set at P < 0.001. [file FEB4-8-481-s006.tiff]
