# Supplementary material for: A standardized fold change method for microarray differential expression analysis used to reveal genes involved in acute rejection in murine allograft models
Source: FEBS Open Bio. 2018 Jan 25;8(3):481–90. doi: 10.1002/2211-5463.12343 (PMC5832988; doi:10.1002/2211-5463.12343)
Supplement: Supplementary file 7 — Table S1. Evaluation of three methods with the level of significance set at P < 0.01. Table S2. Evaluation of three methods with the level of significance set at P < 0.001. Table S3. Primer sequences for qRT‐PCR. [file FEB4-8-481-s007.docx]

**Supplementary Table 1. Evaluation of three methods with the significant p-value under 0.01.**

|  |  | **T-test** | **Limma** | **SFC** |  |
| --- | --- | --- | --- | --- | --- |
|  | **H0** | | | | |
|  | FPR (%) | 0.970 | 1.082 | 1.420 |  |
|  | FNR (%) | 0.000 | 0.000 | 0.000 |  |
|  | Calls in total (%) | 0.970 | 1.082 | 1.420 |  |
|  | **H1 : simulated** **real positive calls = 1%** | | | | |
|  | FPR (%) | 1.379 | 1.134 | 1.244 | θ=10% |
|  |  | 2.689 | 1.258 | 1.007 | θ=25% |
|  |  | 6.099 | 1.685 | 0.729 | θ=50% |
|  | FNR (%) | 13.458 | 38.567 | 12.100 | θ=10% |
|  |  | 1.092 | 15.883 | 0.267 | θ=25% |
|  |  | 0.808 | 5.333 | 0.000 | θ=50% |
|  | Calls in total (%) | 2.224 | 1.733 | 2.104 | θ=10% |
|  |  | 3.644 | 2.080 | 1.987 | θ=25% |
|  |  | 7.023 | 2.608 | 1.714 | θ=50% |
|  | **H1 : simulated real positive calls = 5%** | | | | |
|  | FPR (%) | 5.027 | 1.598 | 0.735 | θ=10% |
|  |  | 18.756 | 4.090 | 0.259 | θ=25% |
|  |  | 38.461 | 9.801 | 0.134 | θ=50% |
|  | FNR (%) | 13.419 | 39.507 | 13.363 | θ=10% |
|  |  | 0.924 | 14.851 | 0.449 | θ=25% |
|  |  | 0.699 | 3.790 | 0.000 | θ=50% |
|  | Calls in total (%) | 9.101 | 4.540 | 5.026 | θ=10% |
|  |  | 22.767 | 8.140 | 5.220 | θ=25% |
|  |  | 41.500 | 14.117 | 5.123 | θ=50% |
|  | **H1 : simulated real positive calls = 10%** | | | | |
|  | FPR (%) | 14.235 | 2.847 | 0.218 | θ=10% |
|  |  | 41.815 | 9.210 | 0.066 | θ=25% |
|  |  | 61.904 | 20.666 | 0.020 | θ=50% |
|  | FNR (%) | 13.950 | 40.375 | 16.286 | θ=10% |
|  |  | 1.096 | 13.775 | 1.193 | θ=25% |
|  |  | 0.652 | 2.9644 | 0.000 | θ=50% |
|  | Calls in total (%) | 21.416 | 8.525 | 8.567 | θ=10% |
|  |  | 47.523 | 16.911 | 9.939 | θ=25% |
|  |  | 65.648 | 28.303 | 10.017 | θ=50% |

**Supplementary Table 2. Evaluation of three methods with the significant p-value under 0.001.**

|  | **T-test** | **Limma** | **SFC** |  |
| --- | --- | --- | --- | --- |
| **H0** | | | | |
| FPR % | 0.107 | 0.116 | 0.337 |  |
| FNR % | 0.000 | 0.000 | 0.000 |  |
| Calls in total (%) | 0.107 | 0.116 | 0.337 |  |
| **H1 : simulated real positive calls = 1%** | | | | |
| FPR % | 0.184 | 0.139 | 0.248 | θ=10% |
|  | 0.558 | 0.141 | 0.151 | θ=25% |
|  | 2.084 | 0.152 | 0.086 | θ=50% |
| FNR % | 23.892 | 97.883 | 18.25 | θ=10% |
|  | 3.592 | 95.867 | 0.833 | θ=25% |
|  | 0.808 | 98.742 | 0.000 | θ=50% |
| Calls in total (%) | 0.938 | 0.159 | 1.057 | θ=10% |
|  | 1.510 | 0.180 | 1.134 | θ=25% |
|  | 3.048 | 0.163 | 1.078 | θ=50% |
| **H1 : simulated real positive calls = 5%** | | | | |
| FPR % | 1.611 | 0.145 | 0.115 | θ=10% |
|  | 8.489 | 0.259 | 0.022 | θ=25% |
|  | 23.793 | 0.463 | 0.009 | θ=50% |
| FNR % | 23.386 | 96.818 | 19.480 | θ=10% |
|  | 3.977 | 97.593 | 1.724 | θ=25% |
|  | 0.700 | 99.583 | 0.000 | θ=50% |
| Calls in total (%) | 5.358 | 0.297 | 4.131 | θ=10% |
|  | 12.862 | 0.367 | 4.930 | θ=25% |
|  | 27.565 | 0.460 | 5.004 | θ=50% |
| **H1 : simulated real positive calls = 10%** | | | | |
| FPR % | 5.472 | 0.211 | 0.020 | θ=10% |
|  | 25.920 | 0.501 | 0.004 | θ=25% |
|  | 48.030 | 0.726 | 0.001 | θ=50% |
| FNR % | 24.009 | 96.539 | 24.819 | θ=10% |
|  | 4.350 | 96.208 | 3.771 | θ=25% |
|  | 0.671 | 99.288 | 0.004 | θ=50% |
| Calls in total (%) | 12.523 | 0.536 | 7.535 | θ=10% |
|  | 32.892 | 0.830 | 9.625 | θ=25% |
|  | 53.160 | 0.725 | 10.000 | θ=50% |

**Supplementary Table 3. Primer sequences for qRT-PCR.**

| **Genes** | **Accession Number** | **Forward (5^’^–3^’^)** | **Reverse (5^’^–3^’^)** |
| --- | --- | --- | --- |
| ***Pdcd1lg2*** | NM_021396 | CTCTACCAGGTCACCAGTGTTCT | GGGTTCCATCCGACTCAGAGG |
| ***Cd8a*** | NM_001081110 | TGCCATGAGGGACACGAATAATAAG | AGTAGAGTTCACTTTCTGAAGGACTG |
| ***Ifng*** | NM_008337 | CCATCAGCAACAACATAAGCGTC | TGTTGACCTCAAACTTGGCAATAC |
| ***Tnfrsf9*** | NM_011612 | CCCTGGACGAACTGCTCTCTAG | CTGGAGTCACAGAAATGGTGGTAC |
| ***Klrk1*** | NM_033078 | GTTTCCTCAAGAGAGGGCTACTGT | AAATTGGTAACAGTTGTTTCTGTGACAT |
| ***Ido1*** | NM_008324 | TATCCAGTGCAGTAGAGCGTCAA | GGTCCACAAAGTCACGCATCC |
| ***Cd27*** | NM_001033126 | GCCAGCGGTCATCCCATAGA | GGCCCGTGGTTTCTTCTTTGA |
| ***Prf1*** | NM_011073 | GCATGTACAGTTTTCGCCTGGT | GTGCGTGCCATAGGAGGAGAT |
| ***Pdcd1*** | NM_008798 | TGACTTCCACATGAACATCCTTGAC | GTCTCCAGGATTCTCTCTGTTACCA |
| ***Ccl3*** | NM_011337 | TGTCATTTTCCTGACTAAGAGAAACC | GTTCCAGGTCAGTGATGTATTCTTG |
| ***Xcl1*** | NM_008510 | TGTGGAAGGTGTGGGGACTG | CCCTCCCAGATGATATAGGTCTTGAT |
| ***Ly6f*** | NM_008530 | GCTGGAGTGCTATAACTGCCTG | ACCTGCTGAGAAATGCAGACTG |
| ***Cst7*** | NM_009977 | GCCTGAAATATATGCTGGAGGTGAA | AATATAGAGTCCGCTTCAAGGCA |
| ***Gzmb*** | NM_013542 | CAAAGGCAGGGGAGATCATCG | TTGATCGAAAGTAAGGCCATGTAGG |
| ***Nkg7*** | NM_024253 | ACAGGTCCTCACTTCTCTGCC | GTCACATGGATATAACCTGCTACTTG |
| ***Ccl4*** | NM_013652 | ATGAAGCTCTGCGTGTCTGC | TCAGAGCCCATTGGTGCTGA |
| ***Crtam*** | NM_019465 | CCATTCAGCTACACAACTCTCCATTA | AAGGAGTCACTAACACGGTCACT |
| ***Tgtp1*** | NM_011579 | TCCCTAAGAGGAAAGCCATCACA | GTATGGTAGAAGCTCAGCAGTGG |
| ***Gbp2b*** | NM_010259 | AGATTGAAATGGAACGTATAAAAGCAGA | ATGTGCTCTTGATAACTCTGTTCCTT |
| ***18S*** | X03205.1 | ATGAGTCCACTTTAAATCCTTTAACGA | CTTTAATATACGCTATTGGAGCTGGAA |

**Supplementary Table 4. GO term and pathway enrichment analysis based on the 52 significant genes.**

Sheet 1: GO term enrichment analysis with three categories BP (biological process), MF (molecular function) and CC (cellular component).

Sheet 2: Pathway enrichment analysis based on the KEGG dataset.
